# Intensity of statin therapy and primary prevention of cardiovascular in Korean patients with dyslipidemia

**DOI:** 10.1097/MD.0000000000037536

**Published:** 2024-03-15

**Authors:** Sun Ok Song, Min Jin Kang, Sunghwan Suh

**Affiliations:** a Division of Endocrinology and Metabolism, National Health Insurance Service Ilsan Hospital, Goyang, Republic of Korea; b Department of Policy Research Affairs, National Health Insurance Service Ilsan Hospital, Goyang, Republic of Korea; c Division of Endocrinology and Metabolism, Samsung Changwon Hospital, Sungkyunkwan University School of Medicine, Changwon, Republic of Korea.

**Keywords:** cardiovascular disease, intensity, primary prevention, statin

## Abstract

This study aimed to investigate the association between the intensity of statin therapy and the development of cardiovascular disease (CVD) and diabetes in individuals without prior diabetes who were being treated for dyslipidemia with statins for the primary prevention of CVD, using the National Health Insurance Service-Health Screening database. The database is a longitudinal cohort study of Korean men and women 40 years of age or older who underwent comprehensive biannual screening health examinations by Korean National Health Insurance Service from January 1, 2002, to December 31, 2015. We included patients in the health screening checkup cohort who underwent health checkups in 2009 and 2010.The primary outcome was the occurrence of a first major cardiovascular or cerebrovascular event, new-onset diabetes. A total of 20,322 participants without prior diabetes at baseline from 2009 to 2015 were followed up for a mean duration of 81.2 ± 6.6 months. The mean age of all participants at baseline was 59.2 ± 8.4 years and 43.0% of them were male. Their index low lipoprotein cholesterol level was 130.4 ± mg/dL, the mean duration of taking statins was 337.4 ± 52.3 days, and 93.9% of them had been taking moderate-intensity statins. At that time, a total of 641 diabetes cases occurred, 41 from using low-intensity statins, 588 from moderate-intensity statins, and 11 from high-intensity statins. The results indicated no significant differences in the incidence of death, CVD death, or CVD among those in the strong statin group compared with the reference groups. While statin treatment for the primary prevention of CVD in patients with dyslipidemia showed a subtle difference in the incidence of diabetes, there was no difference in the occurrence of CVD or CVD death according to statin intensity.

## 1. Introduction

Cardiovascular disease (CVD) presents significant health and economic challenges globally.^[1]^ Low lipoprotein cholesterol (LDL-C) level is a well-established risk factor for CVD, as corroborated by many previous studies.^[2]^ Although there is consensus on the value of lowering LDL-C levels, recommendations on how to do so have shifted over time.^[3]^ The efficacy of statin therapy in reducing LDL-C and subsequently lowering the risk of major coronary events, coronary revascularization, and ischemic stroke is widely acknowledged. Furthermore, it is recognized that the absolute benefits of statin therapy are primarily contingent upon the degree of LDL-C reduction achieved and the underlying risk of vascular disease within the treated population.^[4]^

Statin therapy stands as the pivotal intervention in the prevention of CVD. Numerous large-scale randomized controlled trials have unequivocally demonstrated the profound efficacy of statins in markedly reducing the incidence of subsequent cardiovascular events and mortality across heterogeneous patient cohorts.^[4]^ The clinical merit associated with the reduction of LDL-C levels through statin therapy remains universally acknowledged. This contention is underscored by the findings of the Cholesterol Treatment Trialists’ Collaboration, elucidating that the extent of clinical benefit conferred by statin therapy is commensurate with the absolute decrement in LDL-C levels.^[4]^ Clinical trials have demonstrated that administering high-intensity statin regimens may yield superior efficacy compared to low-intensity statin therapy in reducing future cardiovascular events.^[5–7]^ In addition, the safety and efficacy of high-intensity statins for achieving very low LDL-C levels in patients with CVD was confirmed from substantial meta-analyses.^[4]^ Consequently, the 2013 guidelines established by the American College of Cardiology/American Heart Association (ACC/AHA) emphasized the pivotal role of statin therapy in LDL-C reduction.^[8]^ This transformative paradigm has evoked considerable debate among healthcare professionals who strive to ascertain optimal lipid performance metrics and patient-centered outcomes.^[9]^ However, the Veterans Affairs healthcare system has issued distinct dyslipidemia guidelines advocating for the utilization of moderate-intensity statins as the primary treatment modality for the majority of patients afflicted with cardiovascular disease CVD. These guidelines justify their recommendation by citing insufficient evidence to support the widespread adoption of high-intensity statin therapy, except in select subpopulations characterized by heightened cardiovascular risk.^[10]^ Moreover, a recent cohort study revealed that achieving a LDL-C level of 70 mg/dL or lower did not confer any additional clinical benefit.^[11]^ Consequently, these findings challenge the veracity and universality of current treatment guidelines recommending for very low target LDL-C levels across all patients with established CVD.

The US Preventive Services Task Force reported in 2022 that those with one or more risk factors for CVD or an estimated 10-year risk of a CVD event of more than 10% had a moderate to high net benefit among adults 40 to 75 years old without a history of CVD. It was recommended physicians should prescribe statins for primary cardiovascular disease prevention.^[12]^ The US Preventive Services Task Force 2022 advised moderate-intensity statin therapy and considered it appropriate for primary prevention of CVD in most patients, while the 2016 guideline called for low to moderate-intensity statin medication. The effects of various statins have not been well-compared, and there is a lack of research on Asians, especially. Therefore, our objective was to investigate the incidence of CVD development and mortality according to the intensity of statin therapy in patients with dyslipidemia for primary prevention for CVD. We aimed to discern whether any differences in CVD occurrence and mortality could be attributed to the intensity of statin treatment, using the Korean National Health Insurance Service (NHIS) database.

## 2. Material and methods

All study protocols were approved by the Institutional Review Board (IRB) of Dong-A Medical Center (IRB number DAUHIRB-17-049) and the IRB of the National Health Insurance Service Ilsan Hospital (IRB number NHIMC 2016-03-004 and 2021-03-053) and adheres to the tenets of the Declaration of Helsinki. The National Health Insurance Service-Health Screening Cohort (NHIS-HEALS) data do not contain any identifying information. Due to the retrospective nature of the study, the requirement to obtain informed consent was waived by the IRB because the database was accessed for analysis purposes only, and personal information was not accessed. This study used the NHIS-HEALS data (NHIS-2021-2-081) from the NHIS.

### 2.1. Data source

The national health insurance system in Korea was initiated in 1963 according to the National Health Insurance Act, and it became compulsory for all citizens in South Korea to participate.^[13]^ The NHIS maintains and manages all databases for Korea’s health service utilization currently. The NHIS is also in charge of the national health examination programs, which include a general health examination for all insured employees or self-employed persons over the age of 40 years, as well as their dependents; it is also recommended that this examination be undertaken at least biannually. Moreover, life-transition examinations (for those reaching milestones of 40 and 66 years of age), cancer examinations, and pediatric examinations (for those between 4- and 71-months post-birth) are also managed by the NHIS.

The NHIS constructed the NHIS-HEALS database in 2015. The NHIS-HEALS is a cohort of participants who participated in health-screening programs provided by the NHIS in the Republic of Korea (hereafter Korea). To construct the NHIS-HEALS database, a sample cohort was first selected from the 2002 and 2003 health-screening participants, who were in the age group of 40 to 79 years in 2002 and followed up through to 2013. This database is a longitudinal cohort of a total of 514,866 health-screening participants who comprised a random selection of 10% of all health-screening participants in 2002 and 2003.^[14]^ At the time of the health checkups, systolic and diastolic blood pressures (DBPs) were also measured; serum samples for fasting glucose, hemoglobin, total cholesterol, and LDL-C levels were also obtained after an overnight fast at each examination site. Detailed histories of smoking status, alcohol consumption, and physical activity (including amount and frequency) were obtained using questionnaires. Participants’ socioeconomic status was categorized into 4 groups based on the 10 strata of income levels provided in the NHIS (2002–2010) database.

This cohort included the diagnosis codes in accordance with the International Classification of Diseases 10th revision (ICD-10) as well as outpatient or inpatient status, drug name, dosage, prescription date, duration, and method of administration. The NHIS provided data with individual identifiers removed in accordance with the Act on the Protection of Personal Information maintained by public agencies. The database included an unidentifiable code representing each individual with the patient’s age, sex, medical history, lipid profiles, and a list of prescribed drugs. We used the updated NHIS-HEALS data followed up through 2015.

### 2.2. Study population

**Figure 1.**
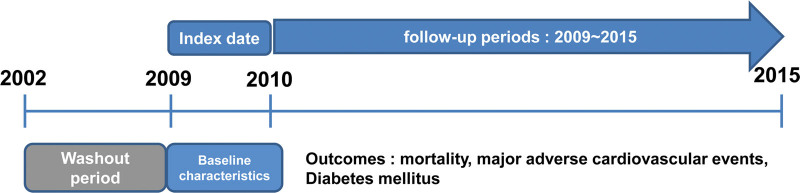
Timeline of the determination of exposure to statin therapy and study outcomes for the study period.

This study enrolled patients with LDL cholesterol levels measured in 2009 or 2010 who had been taking statins until they underwent the lipid profile test. We used the database from 2009 to 2010 as a baseline because the results of LDL-C levels were available from that period (n = 350,041). Patients with changes in the statin type and dose within the previous year were excluded from the analysis. Outlier were also excluded abnormalities exceeding the range from 1 to 99 percentile as follows; waist circumference was from 35 cm to 125 cm, systolic blood pressure were between 60 and 240 mm Hg, DBP were between 40 and 160 mm Hg, hemoglobin between 6 to 20 g/dL, fasting glucose between 40 and 300 mg/dL, total cholesterol between 40 and 400 mg/dL, LDL-C between 29 and 300 mg/dL, triglyceride between 10 and 600 mg/dL, HDL-C between 10 and 200 mg/dL, aspartate transaminase or alanine transaminase level < 200 IU/dL, creatinine level < 2.0 mg per deciliter (176.8 μmol per liter), diabetes, uncontrolled hypertension (systolic blood pressure > 190 mm Hg or DBP > 100 mm Hg). In addition, we excluded individuals diagnosed with cardiovascular or cerebrovascular diseases. Patients with malignant tumors were excluded from the analysis before study entry with preexisting diagnosis of other cancer (C code, C00-C97) according to the ICD-10. Consequently, a total of 23,897 participants were included in our follow-up study. The detailed procedure is shown in Figure 1 (study flowchart). We analyzed the incidences of CVD, diabetes, and mortality.

### 2.3. Operational definition

#### 2.3.1. Use of statin.

Use of statin was defined as receipt of a prescription for the drug during the study period. We only included patients who were prescribed the same dose and type of statin for more than 180 consecutive days, at least during the last 1 year before LDL-C measurement in 2009 and 2010. They were selected as index patients. Our categorization of the LDL-C index was based on an observed achieved value after at least 180 days of statin treatment; however, this level may not have represented a stable LDL-C level over the subsequent years. This observational cohort study compares the risk of mortality and CVD among patients with dyslipidemia without preexisting CVD by observing LDL-C levels after at least 6 months of statin therapy. Patients between the ages of 40 to 79 years with statin treatment were identified. Statin intensity was sorted as High-intensity statin therapies are atorvastatin (40–80 mg) or rosuvastatin (Crestor; 20–40 mg). Moderate-intensity statin therapies include atorvastatin (10–20 mg), rosuvastatin (5–10 mg), simvastatin (20–40 mg), pravastatin (40–80 mg), pitavastatin (2–4 mg), and several others according to the ACC/AHA guidelines 2013.^[8]^

### 2.4. Diagnosis of cardiovascular/cerebrovascular disease

Patients hospitalized with a first diagnosis of CVD were recruited by eliminating those with a prior diagnosis of CVD before 2009. CVD was defined as the occurrence of coronary artery disease (codes I20-25 with documentation of coronary angiography or coronary artery bypass surgery within 7 days of admission), stroke (codes G46, I63-64 with documentation of computed tomography scan or magnetic resonance imaging within 7 days of admission), and/or peripheral artery disease (codes E10-E14 with.5 and I73). Patients who had been hospitalized with a principal discharge diagnosis of CVD^[15]^ were identified using ICD-10 codes.

### 2.5. Diagnosis of diabetes

People who had been diagnosed with diabetes (ICD-10 codes E10–E14), or a fasting blood glucose level of 126 mg/dL or greater.

### 2.6. End point

The primary outcome was the occurrence of the first major cardiovascular or cerebrovascular event, defined as nonfatal myocardial infarction, nonfatal stroke, hospitalization for unstable angina, arterial revascularization procedure, confirmed death from cardiovascular causes, or new-onset diabetes.

### 2.7. Statistical analysis

We used the Cox proportional hazards model to estimate the coefficients and hazard ratios associated with each potential risk factor for the incidence of diabetes, and 95% confidence intervals for the comparison of event rates in each group. The primary analyses were performed on an intention-to-treat basis. Study participation was considered to be complete if any individual participant at the time had an occurrence of the primary endpoint or had been followed through December 31, 2015. The follow-up time was calculated as the time between enrollment and the cardiovascular event, cerebrovascular event, diagnosis of diabetes, date of death, or December 31, 2015, whichever occurred first. We did not consider the interaction terms between independent variables. The times to outcome events were visualized using Kaplan–Meier graphs according to confounding factors. Differences in the Kaplan–Meier curves between the groups were assessed using the log-rank test. All analyses were conducted using the SAS software (version 9.4; SAS Institute Inc., Cary, NC).

## 3. Results

### 3.1. Baseline characteristics of participants

**Figure 2.**
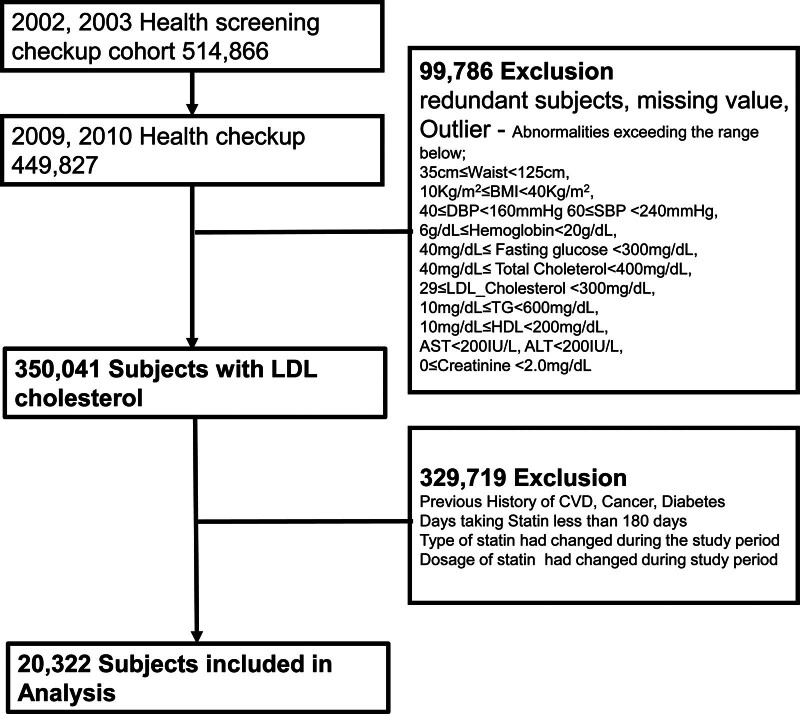
This study flowchart shows the establishment of the study population according to the study inclusion and exclusion criteria.

There were 449,872 patients who were in the health-screening checkup cohort from 2009 to 2010, a total of 514,866 patients underwent Health screening checkups in 2002 and 2003. We excluded individuals who had missing values; any clinical diagnosis of CVD, cerebrovascular disease, cancer, or diabetes; or statin use for <180 days. The study population consisted of 20,322 participants at the baseline who were followed up to December 2015 for a mean duration of 81.2 ± 6.6 months (Figs. 1 and 2 and Table 1). The mean age of all participants at baseline was 59.3 ± 8.4 years and 36.6% of them were male. The baseline demographic characteristics of the study population are presented in Table 1.

**Table 1:** Patient characteristics for achieved low-density lipoprotein cholesterol (LDL-C) groups.

	Total	LDL-C ≤ 70 mg/dL	LDL-C 70.1–100 mg/dL	LDL-C 101.1–130 mg/dL	LDL-C > 130 mg/dL
Number	20,322	1056 (5.2%)	3147 (15.5%)	5844 (28.8%)	10,275 (50.6%)
Age (years, mean, SD) *	59.2 (8.4)	60.9 (8.82)	60.2 (8.8)	59.3 (8.5)	58.7 (8.1)
Sex, male (n, %)*	8743 (43.0%)	607 (57.5%)	1535 (48.8%)	2648 (45.3%)	3953 (38.5%)
Index LDL-C (mg/dL, mean, SD)*	130.4 (36.2)	58.3 (9.7)	87.5 (8.5)	116.6 (8.5)	158.8 (22.5)
Time taking statins (days, mean, SD)	337.4 (52.3)	350.5 (39.7)	343.9 (47.2)	335.3 (53.5)	335.2 (54.0)
Statin potency, No (%)					
High	293 (1.4%)	16 (1.5%)	58 (1.8%)	91 (1.6%)	128 (1.2%)
Mid	19,079 (93.9%)	988 (93.6%)	2911 (92.5%)	5418 (92.7%)	9762 (95.0%)
Low	950 (4.7%)	52 (4.9%)	178 (5.7%)	335 (5.7%)	385 (3.7%)
Statins type, no (%)					
Atorvastatin	13,791 (67.9%)	660 (62.5%)	1983 (63.0%)	3817 (65.3%)	7331 (71.3%)
Rosuvastatin	2185 (10.8%)	132 (12.5%)	353	685 (11.7%)	1015 (9.9%)
Simvastatin	3003 (14.8%)	202 (19.1%)	550	880 (15.1%)	1371 (13.3%)
Others	1343 (6.6%)	62 (5.9%)	261	462 (7.9%)	558 (5.4%)
Follow-up (months, mean, SD)	81.2 (6.6)	81.2 (6.8)	81.2 (6.5)	81.3 (6.1)	81.3 (6.2)

The participants were stratified based on their achieved LDL-C level. For the groups with LDL-C levels >130 mg/dL, 100–130 mg/dL, 70–100 mg/dL, and <70 mg/dL, the mean ages were 58.7, 59.3, 60.2, and 60.9 years, respectively. The average achieved LDL-C levels in each group were 158.8, 116.6, 87.5, and 58.3 mg/dL, respectively. The period of administration of the same dose and type of statin before the achieved LDL-C level was >335 days in each group.

The higher level of the achieved LDL-C had a large proportion of women and younger age individuals. In contrast, a lower index LDL-C level was observed in a larger proportion of men and older individuals. In terms of statin potency, the proportion of moderate-intensity use was >90% in each group, and in terms of component types, the proportion of use among all participants was atorvastatin (67.9 %), simvastatin (14.8 %), rosuvastatin (10.8 %), and other ratios in that order.

### 3.2. Relationship between achieved LDL-C and major adverse cardiovascular events (MACEs)

In the main regression analysis, the adjusted odd’s ratio (OR) for the primary endpoints, including all-cause death, CVD death, coronary heart disease, and stroke by achieved LDL-C groups, are described in Table 2. Of the total 20,322, 386 deaths occurred, 25 (6.5%) were caused by CVD. A total of 438 patients were hospitalized for primary CVD. There were no significant differences in the ORs for all-cause mortality between the groups. There were no significant differences in the ORs for all-cause death, CVD death, or CVD total between the groups, except for total CVD in the group with LDL-C > 130 mg/dL.

**Table 2:** OR of major cardiac events (MACEs) according to achieved LDL-C.

	Total	LDL-C ≤ 70 mg/dL		LDL-C 70.1–100 mg/dL		LDL-C 101.1–130 mg/dL		LDL-C > 130 mg/dL	
	N	N	OR (95%CI)	N	OR	N	OR (95%CI)	N	OR (95%CI)
All-cause death	386	40	1.380 (0.943–2.02)	80	1	99	0.818 (0.609–1.1)	167	0.956 (0.729–1.253)
CVD death	25	4	5.381 (0.984–29.429)	2	1	8	2.509 (0.532–11.832)	11	2.317 (0.51–10.525)
CVD total	438	43	1.240 (0.866–1.775)	98	1	147	0.856 (0.663–1.106)	150	0.537 (0.415–0.694)*

BMI = body mass index, CVD = cardiovascular/cerebrovascular disease, DM = diabetes mellitus, HTN = hypertension, LDL-C = low lipoprotein-cholesterol, MACE = major cardiac events, OR = odds ratio, SES = social economic status.

* Adjusting age, sex, BMI, SES, HTN, DM *P* < .05.

### 3.3. Relationship between statin intensity and MACEs and new onset diabetes

The risks of MACEs and diabetes mellitus according to the statin intensity were analyzed. (Table 3) Compared to the low-intensity statin users, there were no significant differences in the ORs of all-cause death, CVD death, or CVD total between the groups, except for the total CVD in high-intensity statin users (OR 3.688, 95%CI 2.082–6.531). In this study involving over 20,000 participants, only 293 individuals, accounting for a mere 1.4%, were found to have used high-intensity statins for primary prevention of cardiovascular disease (CVD). The utilization of high-intensity statins for primary prevention of CVD is relatively uncommon in cases of CVD total high. However, in real-world practice, it is possible that there may be a subset of individuals with risk factors for CVD that we did not confirm in our claims data, such as smoking, alcohol intake, exercise, family history of CVD, visceral adiposity, and the like.

**Table 3:** OR of major cardiac events (MACEs) and DM development according to intensity of statins.

	Total	Low		Moderate		High	
	N	N	OR	N	OR (95%CI)	N	OR (95%CI)
All cause death	386	21	1	351	1.088 (0.699–1.695)	14	1.954 (0.991–3.853)
CVD death	25	1	1	22	1.228 (0.165–9.159)	2	4.734 (0.426–52.673)
CVD total	438	21	1	390	0.960 (0.619–1.49)	27	3.688 (2.082–6.531)*
Newly diagnosed DM	640	41	1	588	0.725 (0.528–0.995)*	11	0.734 (0.377–1.43)

BMI = body mass index, CVD = cardiovascular/cerebrovascular disease, DM = diabetes mellitus, HTN = hypertension, LDL-C = low lipoprotein-cholesterol, MACE = major cardiac events, OR = odds ratio, SES = social economic status.

* Adjusting age, sex, BMI, SES, HTN, DM *P* < .05.

The risk of new-onset diabetes was significantly lower in moderate-intensity statin users than in low-intensity statin users. (OR 0.725, 95%CI 0.528–0.995). Among all participants in this study, those who used moderate-intensity statins for primary prevention of cardiovascular disease (CVD) accounted for approximately 19,000, representing 93.9%. In most cases, moderate-intensity statins were used for the primary prevention of CVD. The risk of new-onset diabetes was observed to be 27.5% lower in users of moderate-intensity statins compared to users of low-intensity statins. The low-intensity statin users, whom we set as the control group, numbered 950, accounting for 4.7%. The significant difference, with nearly a 20-fold contrast in the number of users between moderate and low-intensity statins, suggests a notable outcome. In reality, among those who used high-intensity statins, totaling 293 individuals (1.4%), there was no significant difference in the risk of developing diabetes compared to the low-intensity statin user population.

## 4. Discussion

In this national study of patients in the Korean NHIS database, we examined a large population-based cohort of patients with dyslipidemia who were taking statins for primary prevention and evaluated the clinical outcomes of CVD as a function of achieved LDL-C levels. We found that an LDL-C level of 70.0 mg/dL or less had no statistically significant association with the risk of CVD compared to patients who had LDL-C between 70.1 and 100.0 mg/dL for primary prevention. We did not find a significant, consistent, or graded association between the intensity of statin therapy and mortality, except for the total risk of CVD in patients receiving high-intensity statins.

With randomized clinical trials (RCTs) focusing on treatment efficacy and safety, evidence to support claims that lower LDL-C levels are associated with clinical benefits remains inconclusive for routine community-based practice.^[11]^ Statins continue to be underutilized in clinical practice despite mounting evidence that they consistently improve the clinical outcomes for the secondary prevention of CVD.^[16]^ While the reasons for this underutilization remain unclear, one contributing factor might be the presumption that higher-intensity statin regimens do not yield superior benefits.^[17]^

The rationale the guidelines for more aggressive treatment of LDL-C levels comes from some RCTs and a meta-analysis of RCTs that compared high-intensity and low-intensity statin treatment.^[4]^ Since the release of the 2013 ACC/AHA cholesterol guidelines,^[8]^ heightened emphasis has been placed on the comparison between high and moderate-intensity statin regimens. The new ACC/AHA cholesterol guidelines^[8]^ for cholesterol management would increase the number of adults eligible for statin therapy, with the increase seen mostly among older adults without CVD.^[18]^ In addition, this recommendation has been reported to overestimate the risk of CVD in Europeans^[19]^ and Asians^[20]^ thereby making it not generally applicable to Asians. Considering the fact that the results of Asian studies were not included in the establishment of the 2013 ACC/AHA guidelines,^[8]^ a study on the benefits and adverse effects of high-intensity administration in Asia, including Korea, is necessary. In this context, the Korean Guidelines for the Management of Dyslipidemia^[21]^ adopted a previous approach instead of following the trends of overseas treatment guidelines. There is an urgent need for studies to evaluate the risk of CVD among the Asians, including the Koreans. In addition, almost all studies on the management of dyslipidemia were conducted in Western countries, and a review of these studies is necessary to determine whether such results are applicable to Asians, including Koreans.

While recent studies have cited that a lower LDL-C level is better,^[22]^ another recent study’s results did not support the blanket principle that a lower LDL-C level is better for all patients in secondary prevention.^[11]^ Thus, “the lower the better” may not be always applicable, but “make it lower with statins” should be always addressed in secondary prevention, even in relatively low-risk patients, including Korean patients. Our study participants were primarily those without a history of CVD. This highlights a fundamental difference between our study and previous studies^[3,17,23]^ which included patients who had already experienced CVD., The primary prevention of CVD with less chronic morbidity, with the potential for fewer events, less disability, and potentially lower expenditures is very important from a socioeconomic perspective. Therefore, this research design in a real-world setting to assess the true value of statin intensity for the primary prevention of CVD would have a great economic impact. We also examined whether the association between achieved LDL-C levels and CVD differed and found no differences in the significance or direction of associations from our main analyses.

The effect of statin intensity on the incidence of new-onset diabetes showed subtle differences but did not depend on the intensity. This difference may originate from the prescription patterns compared to those in Western countries. In contrast to a previous Western study,^[24]^ we observed that moderate-intensity statins were mainly used for the primary prevention of CVD in the study. Each statin differs in terms of absorption, blood protein binding, excretion, and solubility, with variable LDL cholesterol-lowering effects per dose.^[25]^ Many studies on the Asian population have reported the lipid-lowering effects of each statin. Some studies have reported a tendency of higher LDL cholesterol-lowering effects with the same dosage in the Asian population than in the Western populations.^[26,27]^ A recent meta-analysis encompassing 49 trials evaluating 9 distinct interventions to lower LDL-C levels^[3]^ revealed a consistent correlation between absolute decreases in LDL-C levels and reduced relative risks for major vascular events across the various therapeutic modalities. Additionally, a significant linear relationship was observed between attained LDL-C levels and the incidence of cardiovascular outcomes across the spectrum of LDL-C levels investigated.

The most common adverse effects that occur in approximately 4% of statin-treated patients include gastrointestinal disorders, heartburn, and stomachaches. Rare and fatal adverse events such as hepatotoxicity and muscle toxicity can also occur.^[4]^ Furthermore, there are well-documented adverse events such as myalgia, nephropathy, and onset of diabetes mellitus associated with intensified statin treatment that contribute to clinical considerations in statin regimen management.^[28,29]^ In patients aged > 75 years, patients who are taking multiple drugs or drugs with the same metabolic pathway as statins, or those with conditions that require complex medication (e.g., heart transplant or AIDS), statins should be considered at low dosages.^[21]^ In addition, healthcare professionals are concerned about the geriatric-specific adverse effects of high-intensity statins, such as myalgia and potential drug interactions. Ko and colleagues^[30]^ have introduced the concept of the treatment-risk paradox, highlighting that patients at the greatest cardiovascular risk should be treated most aggressively but often do not. Physicians may overemphasize the risk of treatment in the elderly, particularly in the presence of multiple comorbidities.^[31]^ Our findings suggest that high-risk older adults may experience a survival advantage from high-intensity statin therapy. However, adverse drug effects must be considered on an individual basis and integrate them into the risk dialogue between patients and healthcare providers.

In this study, higher LDL-C levels were observed in women and younger age groups. lower cardiovascular disease (CVD) risk in the younger age group and in women, it is possible that the LDL-C targets were adjusted less strictly than with intensive control. Given the circumstance with primary prevention for CVD, while there was a discrepancy with the target LDL values for secondary prevention, it might have been relatively less controlled with lifestyle modifications and follow-up. In addition, administration dose of statin and the of LDL-C could be individualized.

The strengths of this study include the large sample size and the probable inclusion of all hospitalizations for CVD because the NHIS is a compulsory and universal healthcare system in the Republic of Korea. However, this study has some limitations. Medication possession ratios were used as a proxy for adherence; however, as with the NHIS databases, we could only determine if the prescription was dispensed and not if the patient actually took the medication. To ensure the best treatment impact, we included only those patients who were at least 80% adherent to statin therapy during the year prior to the index LDL-C measurement. To inform long-term statin management, we included only patients who had been consistently taking statins for at least 180 days and evaluated the LDL-C levels of patients in community-based care. Because we relied on the ICD-10 administrative codes for the diagnosis of CVD, misdiagnosis and selective misclassification were possible. However, these errors were likely non-differential in the statin intensity group. Furthermore, we were unable to fully adjust for potential confounders that may have affected the results. In addition, we were unable to detect significant differences in mortality according to the intensity of statin therapy, although our mean follow-up duration was 82 months. This study focused on the association between LDL-C levels and primary prevention of CVD outcomes during statin therapy in dyslipidemia patients. We did not evaluate the association between HDL-C levels and the outcomes in detail because LDL-C level is a well-established risk factor for CVD.^[2]^

We evaluated the real-world practice of statin use according to intensity and its association with the development of CVD and all-cause mortality in a national cohort of patients with dyslipidemia. We suggest that low-dose statins could be the first-line therapy for the primary prevention of CVD, given the moderate reductions observed in LDL-C levels, excellent safety profile, demonstrated clinical benefit, and relatively low cost (now that most statins are generic). However, the implications of these results deserve careful consideration in light of the strength of the available trial evidence for different types of therapies. This retrospective study is an important effort to clarify the goals of long-term statin therapy. The findings suggest that targeting an LDL-C level of <100 mg/dL achieves the same cardiovascular risk reduction as more aggressive LDL-C targets, which could help minimize the adverse effects more common with higher statin doses needed for lower LDL targets while maximizing the benefits. The finding of improved outcomes below a threshold LDL-C level also supports the consideration of absolute LDL-C levels instead of relative LDL-C percentage reductions for gauging an adequate response to statin therapy and raises questions about the practice of statin dosing by intensity. This study adds important information to the ongoing discussion on the best statin strategy and LDL-C targets.

In conclusion, while statin treatment for the primary prevention of CVD in patients with dyslipidemia showed a subtle difference in the incidence of diabetes, there was no difference in the occurrence of CVD or CVD death according to statin intensity.

## Acknowledgments

We would like to thank Editage (www.editage.co.kr) for editing and reviewing this manuscript for English language.

## Author contributions

**Conceptualization:** Sun Ok Song.

**Data curation:** Sunghwan Suh.

**Formal analysis:** Sun Ok Song, Min Jin Kang.

**Funding acquisition:** Sun Ok Song.

**Investigation:** Sun Ok Song.

**Methodology:** Sun Ok Song, Min Jin Kang, Sunghwan Suh.

**Project administration:** Sun Ok Song.

**Software:** Min Jin Kang, Sunghwan Suh.

**Supervision:** Sunghwan Suh.

**Resources:** Min Jin Kang, Sunghwan Suh.

**Validation:** Sunghwan Suh.

**Writing – original draft:** Sun Ok Song.

**Writing – review & editing:** Sun Ok Song, Sunghwan Suh.

## References

[1] Writing Group M Mozaffarian D Benjamin EJ. Heart disease and stroke statistics-2016 Update: a report from the American heart association. Circulation. 2016;133:e38–360. 26673558 10.1161/CIR.0000000000000350

[2] Ference BA Ginsberg HN Graham I. Low-density lipoproteins cause atherosclerotic cardiovascular disease. 1. Evidence from genetic, epidemiologic, and clinical studies. A consensus statement from the European Atherosclerosis Society Consensus Panel. Eur Heart J. 2017;38:2459–72. 28444290 10.1093/eurheartj/ehx144 PMC5837225

[3] Silverman MG Ference BA Im K. Association between lowering LDL-C and cardiovascular risk reduction among different therapeutic interventions: a systematic review and meta-analysis. JAMA. 2016;316:1289–97. 27673306 10.1001/jama.2016.13985

[4] Cholesterol Treatment Trialists C Fulcher J O’Connell R. Efficacy and safety of LDL-lowering therapy among men and women: meta-analysis of individual data from 174,000 participants in 27 randomised trials. Lancet. 2015;385:1397–405. 25579834 10.1016/S0140-6736(14)61368-4

[5] Cannon CP Braunwald E McCabe CH. Intensive versus moderate lipid lowering with statins after acute coronary syndromes. N Engl J Med. 2004;350:1495–504. 15007110 10.1056/NEJMoa040583

[6] Cannon CP Steinberg BA Murphy SA. Meta-analysis of cardiovascular outcomes trials comparing intensive versus moderate statin therapy. J Am Coll Cardiol. 2006;48:438–45. 16875966 10.1016/j.jacc.2006.04.070

[7] LaRosa JC Grundy SM Waters DD. Intensive lipid lowering with atorvastatin in patients with stable coronary disease. N Engl J Med. 2005;352:1425–35. 15755765 10.1056/NEJMoa050461

[8] Stone NJ Robinson JG Lichtenstein AH. 2013 ACC/AHA guideline on the treatment of blood cholesterol to reduce atherosclerotic cardiovascular risk in adults: a report of the American College of Cardiology/American Heart Association Task Force on Practice Guidelines. Circulation. 2014;129(25 Suppl 2):S1–45. 24222016 10.1161/01.cir.0000437738.63853.7a

[9] Drozda JP Jr. Ferguson TB Jr Jneid H. 2015 ACC/AHA focused update of secondary prevention lipid performance measures: a report of the American College of Cardiology/American Heart Association Task Force on performance measures. J Am Coll Cardiol. 2016;67:558–87. 26698405 10.1016/j.jacc.2015.02.003

[10] Downs JR O’Malley PG. Management of dyslipidemia for cardiovascular disease risk reduction: synopsis of the 2014 U.S. Department of Veterans Affairs and U.S. department of defense clinical practice guideline. Ann Intern Med. 2015;163:291–7. 26099117 10.7326/M15-0840

[11] Leibowitz M Karpati T Cohen-Stavi CJ. Association between achieved low-density lipoprotein levels and major adverse cardiac events in patients with stable ischemic heart disease taking statin treatment. JAMA Int Med. 2016;176:1105–13. 10.1001/jamainternmed.2016.2751 27322095

[12] Force USPST Mangione CM Barry MJ. Statin use for the primary prevention of cardiovascular disease in adults: US preventive services task force recommendation statement. JAMA. 2022;328:746–53. 35997723 10.1001/jama.2022.13044

[13] Song SO Jung CH Song YD. Background and data configuration process of a nationwide population-based study using the Korean national health insurance system. Diabetes Metab J. 2014;38:395–403. 25349827 10.4093/dmj.2014.38.5.395 PMC4209354

[14] Seong SC Kim YY Park SK. Cohort profile: the National Health Insurance Service-National Health Screening Cohort (NHIS-HEALS) in Korea. BMJ Open. 2017;7:e016640. 10.1136/bmjopen-2017-016640 PMC5623538 28947447

[15] Cannon CP Brindis RG Chaitman BR. 2013 ACCF/AHA key data elements and definitions for measuring the clinical management and outcomes of patients with acute coronary syndromes and coronary artery disease: a report of the American College of Cardiology Foundation/American Heart association task force on clinical data standards (writing committee to develop acute coronary syndromes and coronary artery disease clinical data standards). Circulation. 2013;127:1052–89. 23357718 10.1161/CIR.0b013e3182831a11

[16] Virani SS Woodard LD Akeroyd JM. Is high-intensity statin therapy associated with lower statin adherence compared with low- to moderate-intensity statin therapy? Implications of the 2013 American College of Cardiology/American Heart Association Cholesterol Management Guidelines. Clin Cardiol. 2014;37:653–9. 25324147 10.1002/clc.22343 PMC6649436

[17] Rodriguez F Maron DJ Knowles JW. association between intensity of statin therapy and mortality in patients with atherosclerotic cardiovascular disease. JAMA Cardiol. 2017;2:47–54. 27829091 10.1001/jamacardio.2016.4052

[18] Pencina MJ Navar-Boggan AM D’Agostino RB Sr.. Application of new cholesterol guidelines to a population-based sample. N Engl J Med. 2014;370:1422–31. 24645848 10.1056/NEJMoa1315665

[19] Vaucher J Marques-Vidal P Preisig M. Population and economic impact of the 2013 ACC/AHA guidelines compared with European guidelines to prevent cardiovascular disease. Eur Heart J. 2014;35:958–9. 24569030 10.1093/eurheartj/ehu064

[20] Arai H Sasaki J Teramoto T. Comment on the new guidelines in USA by the JAS guidelines committee. J Atheroscler Thromb. 2014;21:79–81. 24572931 10.5551/jat.ed001

[21] Committee for the Korean Guidelines for the Management of D. 2015 Korean guidelines for the management of dyslipidemia: executive summary (English translation). Korean Circ J. 2016;46:275–306. 27275165 10.4070/kcj.2016.46.3.275 PMC4891593

[22] Cannon CP Blazing MA Giugliano RP. Ezetimibe added to statin therapy after acute coronary syndromes. N Engl J Med. 2015;372:2387–97. 26039521 10.1056/NEJMoa1410489

[23] Natsuaki M Furukawa Y Morimoto T. Intensity of statin therapy, achieved low-density lipoprotein cholesterol levels and cardiovascular outcomes in Japanese patients after coronary revascularization. Perspectives from the CREDO-Kyoto registry cohort-2. Circ J. 2012;76:1369–79. 22447012 10.1253/circj.cj-11-1356

[24] Lavie G Hoshen M Leibowitz M. Statin therapy for primary prevention in the elderly and its association with new-onset diabetes, cardiovascular events, and all-cause mortality. Am J Med. 2021;134:643–52. 33217370 10.1016/j.amjmed.2020.09.058

[25] Smith MEB Lee NJ Haney E. Drug Class Review: HMG-CoA Reductase Inhibitors (Statins) and Fixed-dose Combination Products Containing a Statin: Final Report Update 5. Portland (OR): Oregon Health & Science University; 2009. 21089253

[26] Kwon JE Hyun KY Won SH. Cholesterol lowering effects of lowdose statins in Korean patients. J Lipid Atheroscler. 2014;3:21–8.

[27] Lee E Ryan S Birmingham B. Rosuvastatin pharmacokinetics and pharmacogenetics in white and Asian subjects residing in the same environment. Clin Pharmacol Ther. 2005;78:330–41. 16198652 10.1016/j.clpt.2005.06.013

[28] Dormuth CR Filion KB Paterson JM. Higher potency statins and the risk of new diabetes: multicentre, observational study of administrative databases. BMJ. 2014;348:g3244. 24874977 10.1136/bmj.g3244 PMC4038449

[29] Dormuth CR Hemmelgarn BR Paterson JM. Use of high potency statins and rates of admission for acute kidney injury: multicenter, retrospective observational analysis of administrative databases. BMJ. 2013;346:f880. 23511950 10.1136/bmj.f880

[30] Ko DT Mamdani M Alter DA. Lipid-lowering therapy with statins in high-risk elderly patients: the treatment-risk paradox. JAMA. 2004;291:1864–70. 15100205 10.1001/jama.291.15.1864

[31] Rathore SS Mehta RH Wang Y. Effects of age on the quality of care provided to older patients with acute myocardial infarction. Am J Med. 2003;114:307–15. 12681459 10.1016/s0002-9343(02)01531-0

